# Turbulent kinetic energy from CMR identifies disturbed diastolic flow in myopathic left ventricles

**DOI:** 10.1186/1532-429X-15-S1-E114

**Published:** 2013-01-30

**Authors:** Jakub Zajac, Jonatan Eriksson, Petter Dyverfeldt, Ann Bolger, Tino Ebbers, Carl Johan Carlhall

**Affiliations:** 1 Center for Medical Image Science and Visualization (CMIV), Linköping University, Linköping, Sweden; 2 Department of Medicine, University of California San Francisco, San Francisco, CA, USA

## Background

Turbulent blood flow is a cause of energy loss in the cardiovascular system, and can thus be seen as a measure of flow inefficiency. Novel 4D flow CMR methods permit estimation of intracardiac turbulent kinetic energy (TKE). On the basis of the Reynolds number, one might expect that larger left ventricular (LV) size would promote higher TKE values, and thus lower flow efficiency. In this study, we hypothesized that the TKE of diastolic inflow would be larger in the dilated LVs of heart failure patients compared to normal LVs.

## Methods

**Figure 1:**
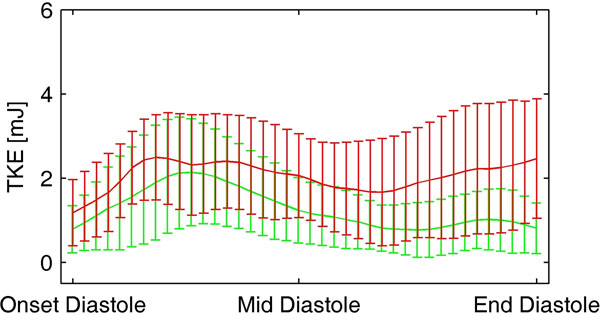
Total LV TKE over diastole (mean±SD). Red: DCM patients, green: healthy subjects.

CMR 4D flow and morphological data were acquired in 9 patients with dilated cardiomyopathy (DCM) (51±13 years old [mean±SD], 5 females) and 11 healthy subjects (Healthy) (43±18 years old., 5 females). The LV was segmented (http://medviso.com/products/segment/) at each diastolic time frame. Total TKE was calculated (Dyverfeldt *et al*., JMRI 2011) within the segmented LV at each diastolic time frame (Figure 1). At early and late diastolic filling, the peak total TKE was compared with transmitral velocity as well as anterior-posterior dimensions of the basal LV and the mitral annulus, respectively (Table 1).

**Table 1:** Correlation between peak total LV TKE and LV diameter, mitral annular diameter, and transmitral peak velocity during early (E) and late (A) diastolic filling.

	TKE vs LV diameter	TKE vs mitral annular diameter	TKE vs transmitral peak velocity
E	r = 0.31	r = 0.56*	r = 0.48*

A	r = 0.62*	r = 0.14	r = 0.39

* p<0.05

## Results

LV end-diastolic diameter and LV ejection fraction were higher and lower, respectively, in DCM compared to Healthy (61±5 vs 47±4 mm, and 41±5 vs 62±3 %, both p<0.001). In the majority of subjects, peaks in total TKE could be observed at early and late diastolic filling. Peak total TKE at late filling was higher in DCM compared to Healthy (2.8±1.7 vs 1.2±0.7 mJ, p=0.01); there was no difference in peak total TKE at early filling between the groups (NS). At early filling, peak total TKE related moderately to mitral annular dimension and transmitral peak velocity (Table 1). At late filling, peak total TKE related moderately to LV dimension (Table 1).

## Conclusions

For both normal and DCM LVs, during early filling TKE relates to inflow characteristics of annular dimensions and mitral inflow velocity, but not to LV size. TKE values in normal LVs are highest during early diastole, and are lower when the LV is at its largest size. In contrast, in DCM LVs TKE increases as the LV size increases, and exceeds values in normal LVs. Energy loss due to late diastolic TKE may reflect inefficient flow in dilated and hypocontractile LVs of heart failure patients.

## Funding

This study was funded by the Swedish Research Council and the Swedish Heart-Lung foundation.

